# Investigating competencies needed by European-trained doctors in rural South African hospitals

**DOI:** 10.4102/phcfm.v12i1.2322

**Published:** 2020-08-25

**Authors:** James R. Barnacle, Oliver Johnson, Ian Couper

**Affiliations:** 1 Department of Infectious Diseases, Imperial College Healthcare NHS Trust, London, United Kingdom; 2 Centre for Health Policy, School of Public Health, University of Witwatersrand, Johannesburg, South Africa; 3 Health Services and Population Research Department, Institute of Psychiatry, Psychology and Neuroscience, King’s College London, London, United Kingdom; 4 Ukwanda Centre for Rural Health, Department of Global Health, Faculty of Medicine and Health Sciences, Stellenbosch University, Stellenbosch, South Africa

**Keywords:** medical education, rural medicine, foreign-qualified doctors, clinical skills, competencies, European-trained doctors

## Abstract

**Background:**

Many European-trained doctors (ETDs) recruited to work in rural district hospitals in South Africa have insufficient generalist competencies for the range of practice required. Africa Health Placements recruits ETDs to work in rural hospitals in Africa. Many of these doctors feel inadequately prepared. The Stellenbosch University Ukwanda Centre for Rural Health is launching a Postgraduate Diploma in Rural Medicine to help prepare doctors for such work.

**Aim:**

To determine the competencies gap for ETDs working in rural district hospitals in South Africa to inform the curriculum of the PG Dip (Rural Medicine).

**Setting:**

Rural district hospitals in South Africa.

**Methods:**

Nine hospitals in the Eastern Cape, KwaZulu-Natal and Mpumalanga were purposefully selected by Africa Health Placements as receiving ETDs. An online survey was developed asking about the most important competencies and weaknesses for ETDs when working rurally. The clinical manager and any ETDs currently working in each hospital were invited to complete the survey.

**Results:**

Surveys were completed by 19 ETDs and five clinical managers. The top clinical competencies in relation to 10 specific domains were identified. The results also indicate broader competencies required, specific skills gaps, the strengths that ETDs bring to South Africa and how ETDs prepare themselves for working in this context.

**Conclusion:**

This study identifies the important competency gaps among ETDs and provides useful direction for the diploma and other future training initiatives. The diploma faculty must reflect on these findings and ensure the curriculum is aligned with these gaps.

## Introduction

The rural district hospital ‘plays a pivotal role in supporting primary health care and … provides level 1 (generalist) services to inpatients and outpatients.’^1^ On-site healthcare provision and organisation of community-based services make these hospitals critical to the delivery of primary health care in line with the principles laid down by the 1978 Alma-Ata Declaration. A key issue in enabling district hospitals to fulfil their critical role is ensuring the competency of their staff, particularly health professionals.

Competency goes beyond technical skills and requires the use of knowledge, clinical reasoning, values, emotions and reflection.^2^ Rural medicine demands a particularly wide range of competencies because of the distinctive quadruple burden of disease in South Africa. This refers to high levels of HIV and TB, maternal and child mortality, injuries, and a rising burden of non-communicable disease. Rural doctors require a broad scope of practice to respond to this need. Expected standards for the competence of healthcare staff working in district hospitals were published by the Department of Health in 2002,^1^ although these focus on overall competence of the health professional team, rather than that of individuals. Several studies have demonstrated the breadth of procedural competencies required by family medicine doctors and medical officers working in district hospitals.^3,4,5,6^

In addition to procedural competencies, broader competencies, such as communication, collaboration and empathy, are universally required for any health professional. These competencies are not used in specific clinical settings, but rather applied with medical knowledge whenever needed in practice. Evidence is poor regarding which broader competencies may be particularly important in rural settings in South Africa, yet it is clear from discussions with rural doctors that broader life and professional skills are indeed essential for their roles.

South Africa has an overall shortage and maldistribution of doctors, both of which are compounded in rural areas. The provincial departments of health, which are responsible for the recruitment of health workers outside of the internship and community service officer programmes, have historically filled some rural posts with foreign-qualified doctors (FQDs). While the exact number of FQDs working in rural South Africa is difficult to determine, 80% of Mpumalanga’s public sector doctors were foreign-qualified in 2011.^7^ Even in peri-urban areas that figure can be as high as 51%.^8^ European-trained doctors (ETDs) make up a proportion of this, attracted by the clinical and surgical learning opportunities,^9^ as well as the unique culture and natural wonders of the country. An increase in interest among British doctors in working in rural South Africa has been driven in part by the re-structuring of training through ‘Modernising Medical Careers’ and the establishment of the non-governmental organisation Africa Health Placements (AHP) in 2005,^10^ as well as growing interest in global health and, more recently, contractual uncertainty for junior doctors in the National Health Service. In addition, since 2015, trainee General Practitioners (GPs) in England and Wales have been able to spend a year working rurally in South Africa as part of a Global Health Fellowship.^11^

The United Kingdom (UK) recognises the personal and organisational benefits of having international experience within its workforce^12,13^ and anecdotally, British doctors have reported gaining communication skills and maturing as professionals during their time in South Africa.^14^ There seems to be a mutual benefit as a flow of doctors helps with rural human resource shortages as well as introducing new ideas to the South African health system. However, Brown et al. caution that the benefits may not always be guaranteed if health workers are not appropriately trained for the roles they fulfil.^15^ Given the anecdotal experiences of British doctors as well as the long list of procedural skills required, to many of which ETDs are not exposed in their training, a significant gap in competencies is likely.

No studies to date have investigated the gap in competencies among ETDs working in rural South Africa but data do exist regarding South African-trained doctors. A study published in 2013 of 60 community service medical officers interviewed on their knowledge and skills revealed the weakest areas were surgical and orthopaedic skills, and anaesthetic and obstetric knowledge and skills.^16^ Internal medicine, paediatrics and family medicine scored most highly on perceived competence.^16^ Other studies have also used self-assessment questionnaires or interviews to investigate skills gaps.^17,18^ A recent study in peri-urban district hospitals in Gauteng indicated that there was no difference in self-reported skills of district hospital doctors based on where undergraduate training occurred.^8^ Over half of the respondents graduated abroad, but 60% of those were from the Democratic Republic of the Congo, and almost half of the study participants had over 10 years’ experience, making this a very different demographic to newly-arrived ETDs.

To address this perceived gap among both local and foreign-trained doctors, the Ukwanda Centre for Rural Health at Stellenbosch University is launching a Postgraduate Diploma in Rural Medicine to equip doctors to work effectively in rural district hospitals. The diploma is structured in two parts over a minimum of 18 months. The first part, for a minimum of six months, involves intense, experiential learning in the context of a regional hospital, with workplace-based supervision and training by accredited specialists, and a focus on clinical competency development. The competencies are structured around three major areas in which procedural skills are needed in rural district hospitals: the emergency department, the labour ward and the operating theatre. The second part, for a minimum of 12 months, involves online learning, regional face-to-face meetings and online discussions while students are placed in a rural district hospital working as medical officers in salaried posts. In these posts they will apply their learning through specific professional assignments with a focus on clinical governance in district health services and delivering healthcare in rural communities. A detailed curriculum for the diploma is still in development. European-trained doctors are expected to make up a proportion of the diploma students and it is crucial that the new diploma meets any gaps specific to European trainees.

The aim of this study was to investigate the gaps in both clinical and broader competencies for ETDs working in rural district hospitals in South Africa. In addition, the study aimed to document how ETDs prepare for such work in South Africa, the strengths they bring with them, and their own ideas for a new training programme. We sought the perspectives of clinical managers from hospitals receiving ETDs, as well as the doctors themselves, with the view that these data would help shape the detailed curriculum of the new diploma.

## Methods

The chosen method was a cross-sectional descriptive study using an online semi-structured questionnaire.

Nine rural district hospitals in KwaZulu-Natal (Bethesda, Estcourt, Manguzi, Mosvold, Mseleni, Tugela Ferry), Eastern Cape (Holy Cross, St. Barnabas) and Mpumalanga (Shongwe) provinces were identified as study sites. These were all the hospitals in which AHP had placed a significant number of ETDs. All ETDs who had been recruited through AHP and were working or had recently worked as medical officers (post-internship generalist doctors) and all hospital clinical managers were invited to participate in the survey. Clinical managers typically have a medical or dental background and oversee the clinical services for an individual hospital.

A survey was designed using SurveyMonkey®, with the introductory page tailored to either ETDs or clinical managers. The questionnaire began by outlining the aims of the study and gaining informed consent for participation. The survey contained 24 questions: eight demographic questions, 10 multiple choice questions prioritising key skills, competencies and knowledge areas in various clinical domains, and six free-text questions, two asking for a ‘top five’ and four open. The focus of the questionnaire was largely skills-based. In order to avoid confusion, the term ‘skills’ was used despite the inclusion of knowledge areas such as ‘paediatric antiretroviral (ARV) regimen’, on the understanding that these focused on application of knowledge in practice and thus included related skills, as well as broader-encompassing ‘competencies’. The skills and domains were derived from the Health Professionals Council of South Africa (HPCSA) intern logbook and a typical South African undergraduate medical programme. Several follow-up emails were sent to all invitees to maximise the response rate.

Results were compiled on a Microsoft Excel® spreadsheet, and frequencies were calculated. The responses to the free-text questions were grouped into similar themes to ease interpretation.

### Ethical consideration

The study was approved by the Health Research Ethics Committee of Stellenbosch University (N17/02/021) and was conducted according to the ethical guidelines and principles of the Declaration of Helsinki.

## Results

Thirty-nine ETDs and nine clinical managers were invited to complete an anonymous survey via email. Twenty ETDs and five clinical managers completed the survey (with response rates of 51% and 56% respectively) between May 2017 and February 2018. Of the 20 ETD respondents, one was excluded owing to incomplete data. The remaining 19 were working as medical officers. All had trained in their country of origin; 14 were from the UK and five from the Netherlands. On average, respondents were 4.5 years post-graduation. Seven of the 19 had a post-graduate qualification in tropical medicine and two of the 19 had a Diploma in HIV Medicine. Six of the doctors had spent less than six months working in South Africa at the time of answering the questionnaire; 13 had spent over six months. Two of the doctors completed demographic data only, leaving 17 respondents for the skills-based questions. All five managers were based in KwaZulu-Natal and had spent an average of 11.2 years working at their hospitals.

The results of ETDs and clinical managers were combined since their responses were similar and the number of clinical managers was small. The most important skills that respondents felt they did not have on arrival were divided into 10 domains (Table 1). Respondents were also asked to choose their ‘top five overall clinical skills’ that they felt they did not have on arrival (Table 2). When asked about particular gaps in a free-text question, the same clinical themes were repeated, as well as three broader competencies: language barrier, ability to work autonomously and adaptation to a new system and culture.

**TABLE 1:** Skills chosen by more than four of the 22 respondents as a ‘top five’ skill in each of the 10 clinical domains. The number of times each skill was chosen by respondents is in brackets.

Domain	Skills (number of times chosen by respondents)							
**General medicine**	Manage HIV and related conditions including antiretroviral therapy (18)	Manage Tuberculosis (15)	Placement of thoracic drainage (8)	Manage Meningitis (Viral, Bacterial, Tuberculous, Fungal) (6)	Lumbar puncture (5)	Lymph node aspiration and cytology (5)	Endotracheal intubation (5)	Central line insertion (5)
**Paediatrics**	Assessment and management of malnutrition (21)	Neonatal resuscitation (11)	Manage and treat dehydration (10)	Paediatric ARV regimen (10)	Paediatric resuscitation (8)	Venesection IV-line insertion, IO infusion (7)	Management of infant feeding problems (6)	
**Obstetrics and Gynaecology**	HIV Mx in pregnancy and delivery and PMTCT (11)	Obstetric US (9)	Foetal distress in labour (9)	Pre-eclampsia (8)	Caesarean section (8)	Hypertension in pregnancy (7)	Management of labour and vaginal delivery (7)	Resuscitation of the newborn (7)
**Mental Health**	Schizophrenia and psychotic disorders (15)	Depression/ bipolar/mood disorders (8)	Assessment of patients in terms of the MHCA (7)	Management of aggressive/ violent patients (7)	The acutely psychotic patient (7)	Alcoholism/ substance abuse (6)		
**General Surgery**	Burns (16)	Suprapubic catheter insertion (11)	Trauma patient (9)	Acute surgical abdomen (7)	Fine needle aspiration (7)	Insertion of intercostal drain (7)	Abdominal US/ peritoneal lavage (6)	Wound care and suturing (6)
**Trauma and Emergency**	Assess, manage and document sexual assault (17)	Manage snake bites (13)	Debride wounds or burns (10)	Insert chest drain (10)	Advanced life support (9)	Intubate and manage airway (8)	Suture laceration (5)	Assess, manage and document interpersonal violence (5)
**Orthopaedics**	Application of POP (14)	Dislocated joint management (12)	Closed reduction of common fractures (9)	Skin traction (8)	Radiology of common conditions (7)	Management of joint injuries (6)	Management of open fracture (6)	Close treatment of common metaphyseal fractures (5)
**ENT and Eyes**	Remove a foreign body from the ear (14)	Drain a peritonsillar abscess (9)	Remove a foreign body from the nose (8)	Manage epistaxis (5)	Remove a foreign body from the eye (5)	Suture an eyelid (5)		
**Anaesthetics**	Spinal anaesthesia (22)	Use of ketamine (21)	Ventilator use (13)	General anaesthesia (10)	Conscious sedation (9)	Use of vasopressors (9)		
**Family Medicine and Primary Care**	Common non-emergency health problems (20)	Undifferentiated problems (12)	Management of poisons and overdoses (12)	Pleural and ascitic taps (10)	Management of STIs (7)	Appropriate intervention in a family crisis (6)		

HIV, human immunodeficiency virus; PMTCT, prevention of mother-to-child transmission; ARV, antiretroviral; IV, intravenous; IO, intraosseous; POP, Plaster of Paris; ART, antiretroviral therapy; US, ultrasound; MHCA, Mental Health Care Act; ENT, Ears, nose and throat; STI, sexually transmitted infection.

**TABLE 2:** Overall top skills in order of frequency chosen by clinical managers (*n* = 5) and doctors (*n* = 17) across all clinical domains (number of times chosen).

Clinical managers	Doctors
Spinal anaesthesia (5)	Spinal anaesthesia (17)
Use of ketamine (4)	Use of ketamine (17)
Assess and manage malnutrition (4)	Assess and manage malnutrition (17)
Common non-emergency health problems/diseases in primary care e.g. HIV/TB/medical conditions (4)	Common non-emergency health problems/diseases in primary care e.g. HIV/TB/medical conditions (16)
Burns (4)	HIV and related including ART (15)
TB (4)	Sexual assault (14)
Application of POP to major joints and limbs (4)	Schizophrenia and psychotic disorders (13)
Remove foreign body from ear (4)	Burns (12)
Manage snake bite (4)	TB (11)
Insert a suprapubic catheter (4)	Undifferentiated problems (11)
Drain a peritonsillar abscess (4)	Manage poisons and overdoses (11)
Manage aggressive patient (4)	

HIV, human immunodeficiency virus; TB, tuberculosis; POP, Plaster of Paris; ART, antiretroviral therapy.

There were some differences in responses between doctors and clinical managers. In the general medicine domain, malaria was selected by three of the five clinical managers as a top five skill, yet none of the doctors selected it. Lymph node biopsy, bone marrow aspiration and trephine, assessment and management of drunken driving, fasciotomy, bone and joint infections, and management of finger and hand injuries were chosen by at least two clinical managers but no doctors. There were four skills that seven or more doctors chose but which none of the clinical managers chose: use of vasopressors, fine needle aspiration, hypertension in pregnancy, and management of mood disorders.

**FIGURE 1:**
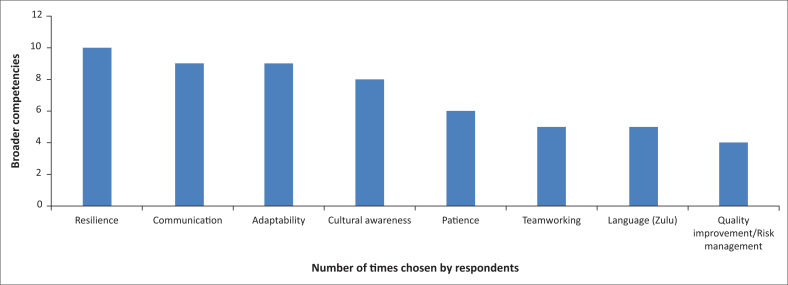
Broader competencies chosen by four or more respondents (*n* = 22).

There was some consistency in the most important broader competencies required when working in rural South Africa, most notably resilience, communication, adaptability and cultural awareness (Figure 1). As well as clinical skills, participants proposed that language lessons, health system orientation, ultrasound training, a mentor system, and a period of shadowing a more experienced doctor would all help prepare and equip newly-arrived doctors for working in rural South Africa. Before arrival, a reading list, online e-learning and better links to existing FQDs working in South Africa were suggested. The key elements respondents chose to include in an academic programme to prepare doctors for working in their hospital, apart from the specific skills, included primary healthcare, dealing with limited resources, simulations, cultural communication skills, referral strategies and health system context.

The relative strengths of ETDs working in South Africa were similarly reported between the clinical managers and doctors. Clinical managers praised a good work ethic, reliability, good record keeping, empathy and interpersonal skills, and stronger knowledge on internal medicine including geriatrics, non-communicable disease and palliative care. These attributes were echoed by the doctors, several of whom also mentioned good use of evidence-based medicine.

## Discussion

This is the first paper specifically to investigate the competency gap in ETDs working in rural South Africa. Clinically, anaesthetics, paediatrics, obstetrics, HIV and TB are areas of weakness. Anaesthetic skills, which are not taught unless in specialist anaesthetic or critical care training in the UK, dominated the skills ranking. Similarly, management of HIV and TB, less common in Europe, were popular choices, and the importance of HIV is also reflected in the frequency that ‘paediatric ARV regimen’ and ‘prevention of mother-to-child transmission’ were selected. Within obstetrics and gynaecology, almost all the key skills were obstetric, although within obstetrics, respondents selected a range of skills, suggesting that obstetrics is generally a weak area. A mortality study of children under five lists prematurity, pneumonia, diarrhoea, birth asphyxia, severe malnutrition and HIV and/or AIDS as the top causes of in-hospital death in Limpopo province.^19^ This helps to explain why malnutrition, dehydration, neonatal and paediatric resuscitation and HIV treatment dominated respondent priorities in paediatrics. The prioritisation of schizophrenia and psychosis in the psychiatry domain may reflect the paucity of psychiatric services^20^ and the fact that mood and anxiety disorders more often go unidentified in rural areas,^21^ meaning that doctors working in rural hospitals are more likely to encounter poorly controlled schizophrenia requiring hospital admission.

Resilience, communication, adaptability and cultural awareness were the most important broader competencies and reflect the challenging environment and significant language and cultural differences faced when working in rural South Africa. These issues are not restricted to ETDs; language is a major barrier to effective healthcare delivery in South Africa in general.^22^ Cultural and linguistic competence is seen as increasingly important in delivering healthcare effectively, especially in such a multi-cultural context as South Africa, and its importance in South African medical schools is recognised.^23^ Respondents in our study did not elaborate on which particular cultural aspects they felt affected their practice. However, patient satisfaction improved following a 10-week isiXhosa course including skills in cultural competence for healthcare workers in the Western Cape^24^ and a similar course in isiZulu or isiXhosa could be included in the six-month part of the diploma. In the absence of a formal teaching course, self-directed online or app-based e-learning, perhaps with a language and culture tutor, either onsite or remotely, would allow doctors to build their skills throughout the 18 months.

Several perceived strengths among ETDs emerged from the study including geriatric medicine and palliative care, as well as generic competencies such as the use of evidence-based medicine and record-keeping. It is important to think of ETDs as possessing a different set of competencies rather than lacking skills, and to build training on the base of existing competencies, while also tapping into their experience to augment local practice.

The results offer insight into how ETDs currently attempt to close the skills gap before coming to South Africa. Of the participating doctors, seven of the 19 had a Diploma of Tropical Medicine and Hygiene (DTMH) and two of the 19 had a Diploma in HIV Medicine. The Global Health Fellowship recommends courses lasting a few days covering trauma and neonatology, such as Advanced Trauma Life Support (ATLS) and Helping Babies Breathe (While R 2019, personal communication, May 28). Africa Health Placement offers weekend courses in infectious diseases and management. None of these courses covers all aspects of practising as a rural doctor in South Africa, and doctors preparing for a placement in South Africa may undertake a selection of these courses piecemeal. Further limitations to these courses is that they are not tailored specifically to South Africa and do not involve on-the-ground experience within a South African healthcare setting.

The findings of this study inform the detailed curriculum content of the Postgraduate Diploma in Rural Medicine (PG Dip [Rural Medicine]) based at Stellenbosch University. This programme recognises that working in a rural district hospital in South Africa demands a specific skillset to manage the range of patients seen and deliver services appropriate to a rural population. It is intended to help both South African and foreign-trained doctors address the shortfall in this skillset. The proposed curriculum reflects some of the core attributes required of doctors specialising in the field of Family Medicine, which differs significantly from the role of primary care doctors in the UK. Family physicians in South Africa are based in rural district hospitals and are thus expected to be competent in the range of procedural skills required of hospitalists, but they are also expected to play a key role in leading and managing community-based primary healthcare. This is done through clinical governance including quality improvement, training, supporting community clinics, advocating and collaborating.^25^ To upscale training of generalist doctors, who can support family physicians in this role, across urban and rural environments, the existing nationally designed Postgraduate Diploma in Family Medicine focuses more on ambulatory primary care.^26^ The PG Dip (Rural Medicine) is not intended for doctors wishing to specialise in Family Medicine, although it could assist towards that, but rather for those who want to build specific competencies for their work in rural district hospitals, regardless of their future career path. Although the questionnaire did not ask respondents how long they planned to spend in South Africa before returning to their country of origin, experience from AHP shows that many ETDs plan to spend a maximum of two years working in rural South Africa before returning to their country of origin. The new diploma reflects this by running a more accelerated programme enabling students to work rurally after six months of training.

The approach taken by the diploma is to address gaps in the competencies of doctors entering the programme. This study informs the focus needed for ETDs who enrol in the diploma, to ensure training meets the needs of rural district hospitals. The experience of intervening towards improved obstetric care through the Essential Steps in Managing Obstetric Emergencies (ESMOE) training programme has shown that practical skills training exercises can improve outcomes, with a 29.3% reported decrease in maternal deaths in areas where ESMOE has been implemented.^27^ It is hoped that the PG Dip (Rural Medicine), by focusing on appropriate competencies for rural district hospitals, can have a similar impact across a broader range of outcome markers. In addition to guiding the diploma, the results of this study can also be used by European doctors preparing for placements in South Africa to identify gaps in their knowledge and address those with courses or private study. Rural district hospitals may also find the study useful to anticipate likely gaps in doctors arriving from abroad, and offer targeted support on arrival. In addition, the GHF and other programmes can use these findings to focus their pre-placement recommendations and courses to outgoing GPs and other doctors. Outside South Africa, these results may be applicable to rural settings in other sub-Saharan African countries and can guide doctors and hospitals elsewhere.

This study has some limitations. Study sites were limited to three South African provinces only. However, these three provinces make up 54% of South Africa’s population.^28,29^ KwaZulu-Natal, where most of our data comes from, is the province with the largest rural population in South Africa. There was an overall response rate of 52% and the absolute number of responses was smaller than anticipated. However, respondents were consistent enough in their choices to be confident that the key skills and themes have been identified. The limited pool of clinical managers of AHP-affiliated hospitals meant that the numbers were always going to be low; input from more managers could have allowed us to analyse the datasets separately, although it is not clear that this would have resulted in any additional insights. This study focuses on the skillset of British and Dutch doctors only. This relates to the fact that, except where government-to-government agreements exist, South Africa does not officially allow recruitment of doctors from other developing countries, as per WHO agreements on international migration of health professionals, and thus AHP’s focus is on recruiting doctors from well-resourced countries. Despite that, South Africa has FQDs from all over the world, and the generalisability of these findings to doctors from other parts of Africa, Cuba or India may be limited, given differences in medical training and postgraduate experience and qualifications, but the principles remain applicable. Finally, we did not collect direct feedback on the structure and content of the post-graduate diploma, which was in the process of being developed at the time of the study, as there were other consultative processes taking place to address these issues.

## Conclusion

We have identified the key gaps in clinical and broader competencies faced by ETDs working in rural district hospitals in South Africa. There is currently no formal programme to address this gap, with doctors relying on on-the-job mentoring by senior colleagues to develop their competencies, which is problematic given staff shortages. The proposed PG Dip (Rural Medicine) can play a meaningful role in addressing the gaps of doctors working in rural district hospitals but its aims and objectives must address these competency gaps and reflect them in its curriculum. The diploma must address broader competencies including linguistic and cultural competency as well as more traditional procedural competencies. The study results will be helpful to doctors planning to work in rural South Africa, and the hospitals welcoming them, to tailor their preparation.
